# Finite Element Analysis of the Non-Uniform Degradation of Biodegradable Vascular Stents

**DOI:** 10.3390/jfb13030152

**Published:** 2022-09-14

**Authors:** Hanbing Zhang, Tianming Du, Shiliang Chen, Yang Liu, Yujia Yang, Qianwen Hou, Aike Qiao

**Affiliations:** Faculty of Environment and Life, Beijing University of Technology, Beijing 100124, China

**Keywords:** biodegradable vascular stents, continuum damage mechanics, finite element method, non-uniform degradation

## Abstract

Most of the studies on the finite element analysis (FEA) of biodegradable vascular stents (BVSs) during the degradation process have limited the accuracy of the simulation results due to the application of the uniform degradation model. This paper aims to establish an FEA model for the non-uniform degradation of BVSs by considering factors such as the dynamic changes of the corrosion properties and material properties of the element, as well as the pitting corrosion and stress corrosion. The results revealed that adjusting the corrosion rate according to the number of exposed surfaces of the element and reducing the stress threshold according to the corrosion status accelerates the degradation time of BVSs by 26% and 25%, respectively, compared with the uniform degradation model. The addition of the pitting model reduces the service life of the BVSs by up to 12%. The effective support of the stent to the vessel could reach at least 60% of the treatment effect before the vessel collapsed. These data indicate that the proposed non-uniform degradation model of BVSs with multiple factors produces different phenomena compared with the commonly used models and make the numerical simulation results more consistent with the real degradation scenario.

## 1. Introduction

In-stent restenosis (ISR) is a major problem limiting the treatment effect of percutaneous coronary intervention [1]. The biodegradable vascular stent (BVS) is the fourth-generation endovascular stent capable of promoting effective vascular remodeling and restoring the elasticity and diastolic–systolic functions of vessels [2]. The BVSs have a greater potential for avoiding ISR than permanent ones [3,4,5].

The BVSs function immediately after they are implanted. The degradation of BVSs involves multiple disciplines such as electrochemistry, metal physics, thermodynamics, materials science, and mechanics [6,7]. The corrosion mechanism of BVSs includes but is not limited to uniform corrosion, pitting corrosion, intergranular corrosion, stress corrosion, and fatigue corrosion [8]. Compared with in vivo and in vitro research, in silico research is widely used in the preliminary study of medical implant developments because it can fully capture the degradation behavior of bioabsorbable materials and the advantages of low cost and time saving [9]. Continuum damage mechanics (CDM) is a common phenomenological approach used to simulate material corrosion for the above-mentioned mechanisms in the corrosive environment under the finite element analysis (FEA) framework.

The uniform degradation of BVSs from the global corrosion that occurs at the entire free surface exposed to a corrosive environment. Uniform corrosion is the most common global corrosion which was mostly used in early studies to simulate stent degradation. Uniform corrosion is the overall thinning caused by chemical and electrochemical reactions on the surface, with an even corrosion rate. The uniform corrosion was simulated using the FEA by uniformly removing elements from BVSs’ outer surfaces [10]. Further, modifications were made to the uniform corrosion model by using arbitrary Lagrangian–Eulerian adaptive meshing [11].

The non-uniform degradation of BVSs from the local corrosion that occurs at specific spots on the surface seriously affects the therapeutic effect [12]. BVSs’ mechanical strength and geometry change during the degradation [13]. The rapid structural weakening caused by local corrosion may lead to adverse events such as fast restenosis due to vessel collapse [14,15].

Stress corrosion, the corrosion under the combined action of tensile stress and blood, is one of the key causes of non-uniform degradation and early failure of BVSs [16]. The stent may crack perpendicular to the stress direction under the safe load. The residual stress after the stent implantation and the working stress during service are the driving stresses of stress corrosion [17]. The CDM method analyzes the stress, strain, and induced damage process of the stent material from the perspective of continuum mechanics [12]. A CDM-based degradation model coupled the stress corrosion model with the uniform corrosion adapted from the work of da Costa-Mattos et al. was established [18,19]. The initial results of the simulations were considered phenomenologically consistent with experimental observations. This coupled formulation was further studied and completed in vitro corrosion tests on two BVSs designs and compared results against simulated predictions for the same designs [20]. The stress corrosion threshold changed dynamically with the material properties and mechanical properties during degradation. However, this issue was not considered in previous studies.

Pitting corrosion is local corrosion caused by the surface defects during stent manufacturing and the stress concentration during angioplasty [8]. A model by expanding on the CDM uniform corrosion element removal model through the inclusion of the assignment and transfer of pitting parameters to capture the effects of pitting corrosion, and results showed agreement with the tests [10]. A physically based pitting corrosion model was established to consider the multi-physical process of magnesium stent degradation, including the transport of magnesium ions. The gaps between phenomenological and physical corrosion models of absorbable mental stents could be addressed [21]. However, the influencing factors of pitting corrosion were studied independently in most previous research, and there is a lack of studies that combine pitting corrosion with uniform corrosion and stress corrosion.

Mass transfer and flow shear stress due to blood circulation can accelerate corrosion (including local and global corrosion etc.). Flow shear stress increased the average uniform corrosion rate, the coverage and depth of local corrosion, and the removal rate of corrosion products from pits [22]. The effect of blood flow should be considered for BVSs degradation.

The BVSs’ edges and corners are susceptible to corrosion, and one example is that the corners would be rounded [23,24]. The damaged edges and corners would change the cross-sectional shape and mechanical properties of the BVSs, which in turn affects the degradation. In the FEA framework, the elements at the edges and corners have more surfaces exposed to the corrosive environment. A uniform and stress corrosion model considering multi-dimensional effects on up to three exposed surfaces was developed and was applied in degradation simulations of biodegradable magnesium alloy stents [25]. As the number of exposed surfaces changes dynamically during the degradation, more exposed surfaces should be considered. A multi-dimensional pitting corrosion model that takes the number of exposed surfaces of the element and the interaction of adjacent elements into account was established and the proportional link between the corrosion rate and the number of exposed surfaces was revealed in the results [26]. However, stress corrosion and the interaction between the stent and the vessel were not considered. 

Iron (Fe), magnesium (Mg) and their alloys have been extensively studied as BVS metals. Both Mg and Fe are essential trace elements in the human body. Mg exhibits excellent biocompatibility, low thrombogenicity, and is critical in many cellular functions [27]. However, the inherently rapid corrosion of Mg in physiological environments is a major concern [28]. Fe and its alloys possess high radial strength, allowing the use of stents with substantially thinner struts and thereby reducing the restenosis rate [29]. The degradation of iron takes too long to be fully degraded over its expected service period and it produces a relatively large volume of iron oxide products which might not be safely metabolized in the body [30,31]. Despite much research that has been completed to tailor the properties of Mg and Fe through alloying, advanced processing, and manufacturing routes, further improvements are still needed to identify an ideal stent material [32,33,34]. In 2013, zinc (Zn) was introduced as an alternative to Mg and Fe, mainly due to its moderate corrosion rate in simulated body fluid [29,35]. Potential toxicity and biocompatibility aspects are important for medical implants [36]. Zinc alloys are biocompatible with acceptable or zero cytotoxicity [37]. As one of the necessary trace elements for humans, zinc can reduce the inflammatory reaction after stent implantation and promote the regeneration of the vascular intima [38]. Therefore, zinc alloys could be used to manufacture BVSs [39].

This paper aims to establish an FEA model for the degradation of zinc alloy stents by simultaneously considering the combined effects of such factors as the number of exposed surfaces in the blood flow, the dynamic changes of material properties, and the addition of pitting corrosion, which may lead to non-uniform degradation. The degradation process and service performance are explained by the phenomenological description of the damage to the BVSs. The corrosion feature of this model will be revealed by comparing the influences of the different factors herein.

## 2. Materials and Methods

The relationship between the number of exposed surfaces and the corrosion rate was considered, and the stress corrosion threshold of an element was dynamically adjusted with the degradation process. Pitting corrosion was combined with uniform corrosion and stress corrosion. The non-uniform degradation of the BVSs under the combined action of multiple corrosion factors was simulated by setting the material properties and corrosion properties for individual elements under the FEA framework. The service performance and support time of the degradable stent were investigated.

### 2.1. Stent Degradation Model

Based on the CDM principle, the macroscopic damage and strength weakening effect of the stent were simulated by establishing a scalar field composed of the damage variable *D* of each element. The damage variable *D* was a gradually cumulative and monotonically increasing function. The effective stress during the damage process can be calculated with Equation (1).

(1) $$\sigma =\bar{\sigma }(1-D)$$

where *σ* is the effective stress, $\bar{\sigma }$ is the undamaged stress, and *D* is the damage variable which increases monotonously from 0 to 1. Linear attenuation adjustment was made to the material properties during degradation through *D* [18]. When *D* equals 0, the material is undamaged, while when *D* approximately equals 1, the material is completely damaged, and the element is removed from the model.

The occurrence of uniform corrosion is only judged by whether the element is at the corrosion surface regardless of the stress state of the stent. An electrochemical reaction of zinc and other metals in the stent with electrolytes in the blood occurs on the initial corrosion surface. The reaction between corrosion products and the blood further exposes the zinc alloy to the blood environment, thus updating the corrosion surface. For the exposed element located on the corrosion surface, the evolution equation of the uniform corrosion damage parameter $D_{U}\;$ can be described with Equation (2) [18].

(2) $$\dot{D_{U}}=dD_{U}=\frac{\delta_{U}}{L_{e}}k_{U}dt$$

where $k_{U}$ is a kinetic parameter related to the uniform corrosion process, $\delta_{U}$ is a characteristic dimension of the uniform corrosion process, and $L_{e}$ is the characteristic length of a finite element. Based on the immersion test in our previous research, the average degradation rate from 0 to 42 days was 0.188 mm/y, which is very close to the degradation rates of zinc alloys with different compositions in other literature [40]. The detailed kinetic parameters $k_{U}$ and $\delta_{U}$ of uniform corrosion are obtained from references and listed in Table 1 [17,18].

For the elements exposed to the corrosive environment during the degradation, $k_{U}$ was dynamically adjusted in proportion to the number of surfaces exposed to the corrosive environment [25], so as to speed up the degradation of the element at the edges and corners of the stent. Considering the convective–diffusion effect of wall shear stress generated by blood flow on stent corrosion products, only the corrosion effect of blood on stent was considered in the current model.

Stress corrosion is relevant to the stress state during the degradation. The stress corrosion threshold is the stress level at which the material can resist crack extension in the corrosive environment. The occurrence of stress corrosion was judged by comparing the equivalent stress $\sigma_{eq}^{*}$ with the stress corrosion threshold $\sigma_{th}$. The maximum principal stress of the element was chosen as the $\sigma_{eq}^{*}$ in this study. The evolution equation of the element’s stress corrosion damage parameter $D_{SC}$ is described with Equations (3) and (4) [18].

If $\sigma_{eq}^{*}\;$<${\;\sigma }_{th}$:

(3) $$\dot{D_{SC}}=0,$$

if $\sigma_{eq}^{*}\;$≥${\;\sigma }_{th}\;$> 0:

(4) $$\dot{D_{SC}}{=dD}_{SC}=\frac{L_{e}}{\delta_{SC}}{\left(\frac{{S\sigma }_{eq}^{*}}{{1-D}_{SC}}\right)}^{R}dt,$$

where $\delta_{SC}$ is a characteristic dimension of the stress corrosion process, *S* and *R* are the functions of the corrosive environment related to the kinetics of stress corrosion. Based on the da Costa-Mattos’ research [19], *S* and *R* are constants due to the constant pH in human’s corrosive environment. $\sigma_{th}$ is closely related to the combination of material composition, metallurgical conditions, and corrosive environment, and usually ranges from 30% of the yield stress to 90% of the ultimate tensile stress [41]. The stress corrosion threshold was set to 30% of the yield stress $\sigma_{s}$ of zinc alloy to ensure safety due to a lack of research on such materials at present. The details of these parameters are listed in Table 1.

The material properties including Young’s modulus and yield stress of the BVSs change with the corrosion [6]. Considering the complexity of corrosion products and uncertainty in stress corrosion experiments, the $\sigma_{s}$ was dynamically adjusted by *D* during degradation to objectively describe the constitutive relationship of the material based on the work of our previous study [17].

As random local corrosion, a pitting corrosion parameter $\lambda_{e}$ was randomly assigned to the element. The evolution equation of the pitting damage parameter $D_{P}$ was described with Equation (5) [10]. The pitting corrosion parameter was transferred to its adjacent elements when an element was removed from the model as shown in Equation (6).

(5) $$\dot{D_{P}}{=dD}_{P}=\frac{\delta_{U}}{L_{e}}\lambda_{e}k_{U}dt,$$

(6) $$\lambda_{e}{=\beta \lambda }_{n},$$

where $\lambda_{n}$ is the pitting parameter of the removed element and β is a dimensionless parameter that controls the acceleration of pit growth [10]. Then the growing of pitting pits was described. The details of these parameters are listed in Table 1.

The above three types of corrosion occur independently. A linear superposition of different scalar fields from different degradation processes was assumed [18]. The total damage variable *D* was the linear algebraic sum of each corrosion damage parameter as shown in Equation (7).

(7) $$D=D_{U}+D_{SC}+D_{P}.$$

**Figure 1:**
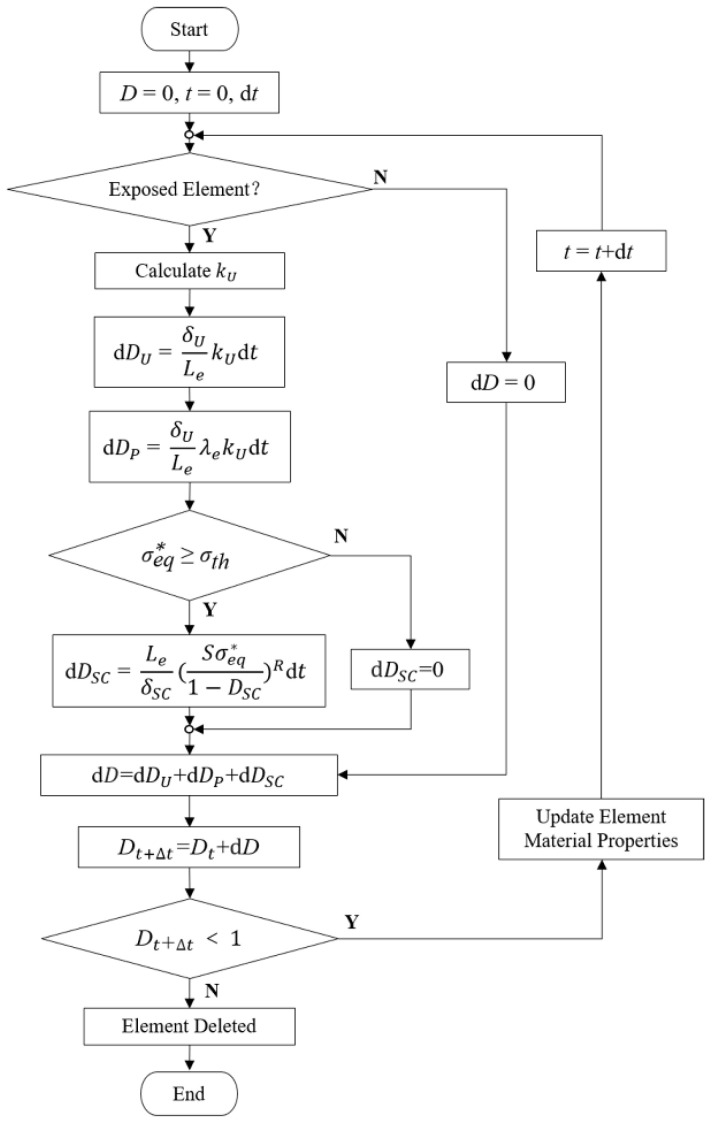
Calculation of damage variables and update of material properties for individual elements.

Further, a dynamic degradation model considering uniform corrosion, stress corrosion, and pitting corrosion was established (Figure 1). The characteristic length $L_{e}$ of the elements was introduced in the evolution process of the above three corrosion mechanisms so that the damage variable *D* could reflect the damage “per unit element”. The influence of the mesh size on the accuracy of the stent degradation model could be avoided to ensure the spatial synchronization between the model and the actual degradation.

### 2.2. Geometry Model

**Figure 2:**
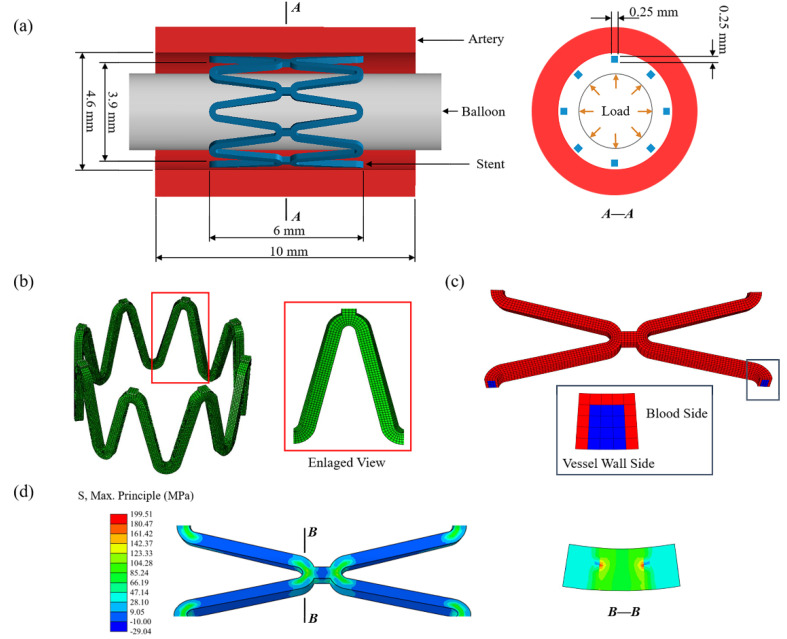
(**a**) The finite element model of the artery, stent, and balloon; (**b**) the mesh of the stent with an enlarged view of the stent strut; (**c**) the initial corrosion surface of the stent; (**d**) the residual stress of the stent after deployment.

The FEA model was composed of an artery, two sets of the eight-corolla stents, and a balloon (Figure 2a). The stent has a thickness of 0.25 mm with its structs, and the artery has an inner diameter of 4.6 mm and a thickness of 1 mm [17]. The artery length was chosen according to three ring lengths to keep a similar artery/ring length ratio of about 1.5 [17]. The balloon has a length of 12 mm and a diameter of 3mm. No stenosis was added to the vessel as this research only focused on stent degradation. Using the Bottom-Up meshing technique, the quadrilateral mesh was divided on the inner surface of the stent, and properly densified the mesh in the corolla; then the stent thickness was offset externally (Figure 2b). The stent meshed with 68,016 nodes and 47,360 C3D8R elements with a characteristic length of 50 μm based on the mesh convergence study.

The stent was modeled as a homogeneous, isotropic, and elastoplastic material. The material properties of the zinc alloy used in this study were measured by a tensile test [18]. The value of 74,300 MPa as Young’s modulus of the undamaged zinc alloy was obtained by tests and the value of 300 MPa was assigned for the completely damaged zinc alloy and linearly decayed over this range by *D* during degradation (Table 2). The balloon was modeled as a cylindrical membrane with a thickness of 0.02 mm. The vessel was modeled as an incompressible material described by a third-order Ogden isotropic hyperelastic material model to simulate its highly nonlinear mechanical [42].

### 2.3. Transfer of Corrosive Properties

All three types of the above-mentioned corrosion only occur on the stent surface in contact with the corrosive environment for alloy-based BVSs. The corrosion surface is continuously updated with the degradation. The element on the corrosion surface has its own corrosion properties, including the exposure property of whether it is in direct contact with the corrosive environment, the number of exposed surfaces, and the pitting corrosion parameter. The corrosion properties will be transferred to the adjacent elements after a complete corrosion element is removed.

The definite location between elements facilitates the identification of corrosion surface and the update of exposure-related corrosion properties of the elements during degradation. The removal of an exposed element only changes the corrosion properties of its face-adjacent elements. The transfer of corrosion properties includes updating the exposure property and increasing the number of exposed surfaces for the face-adjacent elements of the removed element and passing the pitting parameters to them.

The serial number of the element and corresponding node information was obtained after meshing, and the adjacent relationship between elements was obtained by calculating the number of shared nodes through the program. For the C3D8R element used in this research, the element located inside ha six face-adjacent elements, otherwise, it is on the surface. The initial corrosion surface of the stent was set as the elements in contact with blood by identifying the contact pairs between the stent and the vessel after implantation and excluding the surface elements only in contact with the vessel wall (Figure 2c). Then the number of exposed surfaces for the elements located on the initial corrosion surface was counted, and pitting corrosion parameters were assigned by using the Weibull random-number generator. Initial corrosion properties of the elements and adjacent relationships between elements were saved in external files.

### 2.4. Finite Element Analysis

The finite element analysis was divided into two parts: stent deployment and stent degradation. Firstly, the stent deployment was simulated by expanding the stent to 1.1 times the diameter of the vessel by applying displacement to the inner surface of the balloon (Figure 2a). The process was achieved by finite element analysis of the structural mechanics of the stent-vessel model with the Abaqus 6.14/Explicit solver (DS-SIMULIA, Providence, RI, USA) due to its superior contact enforcement method. The balloon was removed from the artery after stent deployment. The residual stress of the stent after deployment acted as the initial stress driving stent degradation (Figure 2d). The corrosion at this stage could be ignored as the stent deployment is short compared to the degeneration process. The general contact was set between each part, the coefficient of friction was assumed to be 0.2 for the tangential contract, and the normal contact was set as the default ‘‘hard” contact [10]. The constraint at axial and circumferential was applied at both ends of the artery to simulate the pull of the surrounding artery. The average ratio of kinetic energy to internal energy was maintained below 5% to minimize the effects of dynamics and ensure that the analysis process is quasi-static.

**Figure 3:**
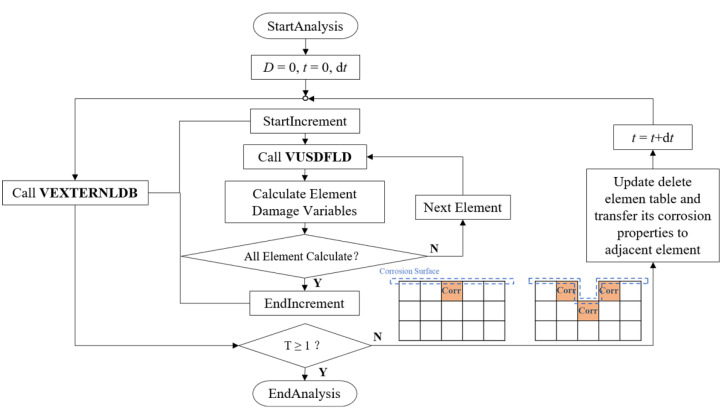
The stent’s degradation was achieved through VUSDFLD and VEXTERNALDB subroutines in ABAQUS/Explicit.

The continuous damage evolution process of zinc alloys was simulated by using Abaqus/Explicit solver and two user-defined subroutines of VEXTERNALDB and VUSDFLD. VEXTERNALDB was used for reading and writing external files in Explicit analysis. The damage variable functions at each integration point of the element in the stent model were defined in the degradation process, and these functions were calculated by VUSDFLD. Corrosion properties of the removed elements in the increment were transferred according to the adjacent relationship of the elements while returned at the end of each increment (Figure 3).

The remained volume of the stent decreased as the element was gradually removed during the degradation. The corresponding time and the volume of the removed elements were the outputs in this process. The volume of the removed element was recorded as the cube of the characteristic length *L_e_*. The mass loss during stent degradation is shown in Equation (8).

(8) $$\mathrm{mass}\;\mathrm{loss}=\frac{\sum \rho \cdot L_{e}{}^{3}}{\mathrm{initial}\;\mathrm{mass}\;\mathrm{of}\;\mathrm{the}\;\mathrm{stent}}\times 100\%,$$

The supporting performance is an important indicator for evaluating the service performance of degradable stents. It expresses effective support time and loss of mechanical strength of the stent. The vessel recoil during degradation is described in Equation (9).

(9) $$\mathrm{vessel}\;\mathrm{recoil}=\frac{\;\mathrm{vessel}\;\mathrm{diameter}\;\mathrm{during}\;\mathrm{degradation}-\mathrm{vessel}\;\mathrm{diameter}\;\mathrm{before}\;\mathrm{stent}\;\mathrm{development}\;}{\mathrm{vessel}\;\mathrm{diameter}\;\mathrm{after}\;\mathrm{stent}\;\mathrm{development}-\mathrm{vessel}\;\mathrm{diameter}\;\mathrm{before}\;\mathrm{stent}\;\mathrm{development}}\times 100\%,$$

The following five circumstances were simulated: (I) Case *U*: uniform corrosion, with constant corrosion rate; (II) Case *U_surf_*: uniform corrosion, with the corrosion rate adjusted according to the number of exposed surfaces; (III) Case *U_surf_SC*: add stress corrosion based on Case *U_surf_*, with constant stress corrosion threshold; (IV) Case *U_surf_SC_th_*: add stress corrosion based on Case *U_surf_*, with dynamic change of stress corrosion threshold according to the corroded status; (V) Case *U_surf_SC_th_P*: add pitting corrosion based on Case *U_surf_SC_th_*. Six simulations on different pitting corrosion distribution locations were conducted to eliminate the randomness of pitting corrosion.

## 3. Results

### 3.1. Effects of the Number of Exposed Surfaces on Stent Degradation

**Figure 4:**
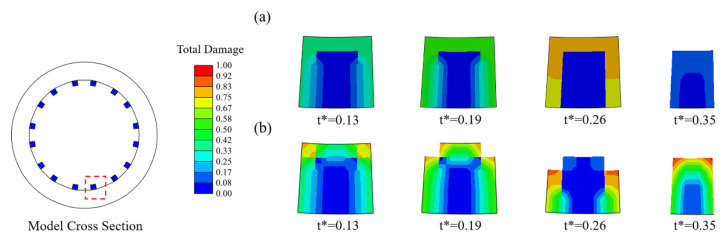
Changes in the cross-section of struts during degradation. (**a**) Case *U*; (**b**) Case *U_surf_*.

In Case *U* (Figure 4a), the damage variables were nearly identical for all exposed elements, and thus the stent struts became thinner due to the simultaneous removal of the elements on the corrosion surface of the stents. In Case *U_surf_* (Figure 4b), the elements at the edges of the stent degraded first as their damage variables were greater than others due to the two surfaces being exposed to the corrosive environment. Subsequently, the number of exposed surfaces of its face-adjacent element increased, and the degradation accelerated with the increase in the rate of damage variable accumulation. The originally square cross-section first became round and further became thinner.

**Figure 5:**
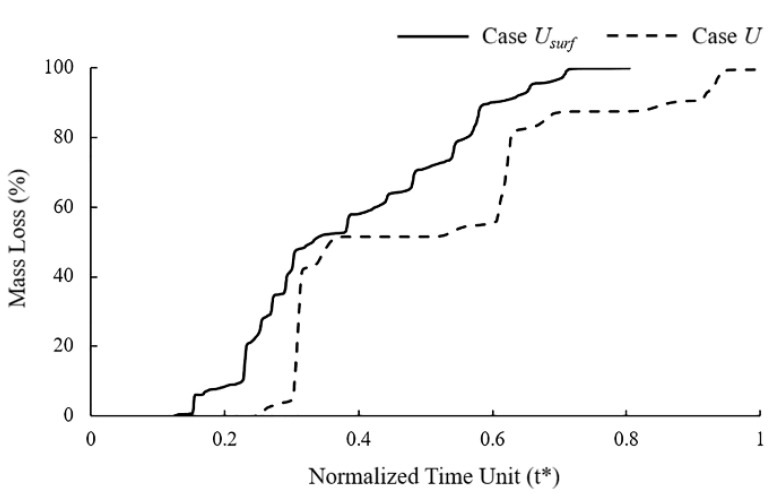
Mass loss of Case *U* and Case *U_surf_* during stent degradation.

It can be observed in the mass loss curve that the the dynamic change of the corrosion rate with the number of exposed surfaces accelerated the time to complete degradation of the stent by 25% (Figure 5). The “stair” caused by the simultaneous removal of elements on the corrosion surface can be observed in the mass loss curve of Case *U*. Elements with approximately the characteristic length accumulated the same damage and were removed from the model simultaneously due to the consistent corrosion rate. The corrosion surface area of the stent became smaller with the degradation, and the height of the “stair” in the curve became lower. The mass loss curve of Case *U_surf_* showed a gradual increase without the obvious mutation in Case *U*.

### 3.2. Effects of Dynamic Changes of Stress Corrosion Threshold on Stent Degradation

**Figure 6:**
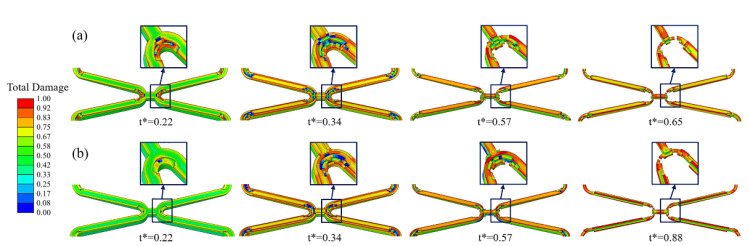
The degradation form of the stent until fracture. (**a**) Case *U_surf_SC_th_*; (**b**) Case *U_surf_SC*.

As local corrosion, stress corrosion occurs in a small area but can greatly impact the structure. Comparing the simulation results of Case *U_surf_SC_th_* and *U_surf_SC*, it can be found that Case *U_surf_SC_th_* mainly showed the fracture at the corolla of the stent (Figure 6a), and Case *U_surf_SC* mainly showed the uniform thinning of the struts (Figure 6b). The degradation of stents in both cases first occurred in the high tensile stress area inside the corolla, and the struts gradually became thinner under the dominant effect of uniform corrosion in the subsequent degradation. The struts of Case *U_surf_SC* were thinner than those of Case *U_surf_SC_th_* when the stent fractured.

**Figure 7:**
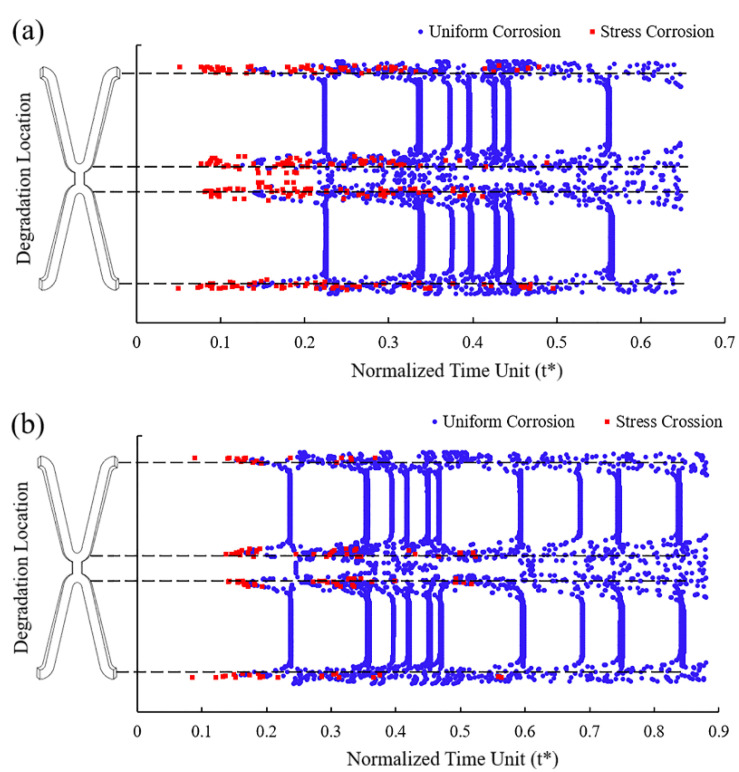
The location of the removed elements and the dominant factors leading to degradation. (**a**) Case *U_surf_SC_th_*; (**b**) Case *U_surf_SC*.

In Case *U_surf_SC_th_* and *U_surf_SC*, the removed elements under the dominant action of stress corrosion were mainly distributed in the high tensile stress area at the corolla of the stent, while those under the dominant action of uniform corrosion were in various areas of the stent (Figure 7). Figure 7a shows that more elements were removed under the dominant effect of stress corrosion as the dynamic reduction in the stress corrosion threshold made the element subject to stress corrosion due to the tensile stress reaching the threshold. The dynamic change of stress corrosion threshold accelerated the degradation of the high tensile stress area and thus sped up the stent fracture.

**Figure 8:**
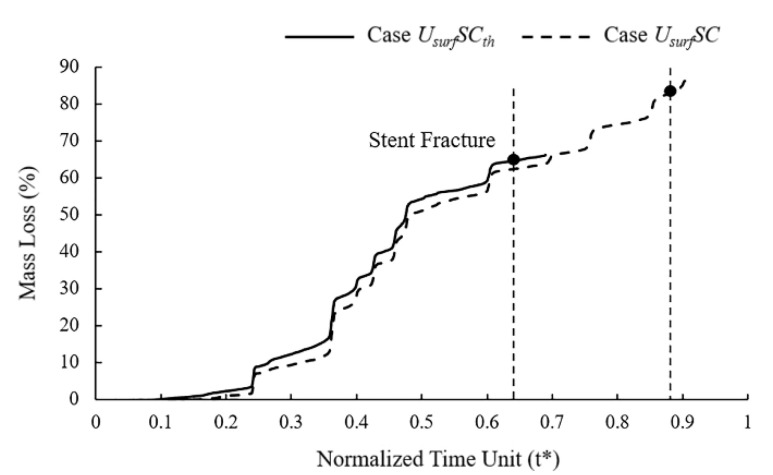
Mass loss of Case *U_surf_SC_th_* and Case *U_surf_SC* during stent degradation.

In mass loss curves (Figure 8), the tendency of Case *U_surf_SC_th_* and *U_surf_SC* were roughly the same, while the stent fracture in Case *U_surf_SC_th_* was earlier than that in Case *U_surf_SC*. The mass loss of the stent increased slowly in the early stage of degradation, and the change was more obvious in the later stage. The mass loss curve showed noticeable changes when the element on the edges or other elements on the corrosion surface were removed simultaneously. The mass loss of Case *U_surf_SC_th_* and *U_surf_SC* were about 65% and 83%, respectively, when the stent fractured. The dynamic reduction in the stress threshold could accelerate the time of stent degradation by 26% and reduce mass loss by 22% when the stent fractured.

### 3.3. Effects of the Addition of Pitting Corrosion on Stent Degradation

**Figure 9:**
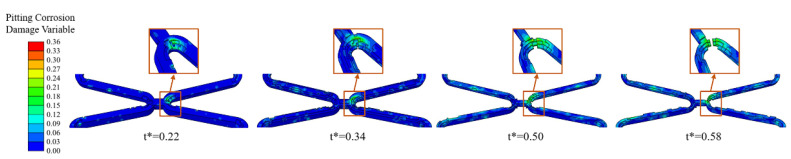
The degradation form of the stent until fracture of Case *U_surf_SC_th_P* when the pitting corrosion occurs at critical locations of the corolla.

Pits caused by pitting corrosion can be seen during the degradation and would develop further over time. The addition of the pitting corrosion model accelerated the degradation. Especially when the pitting corrosion occurs at critical locations of the corolla, the local fracture would be obviously accelerated (Figure 9).

**Figure 10:**
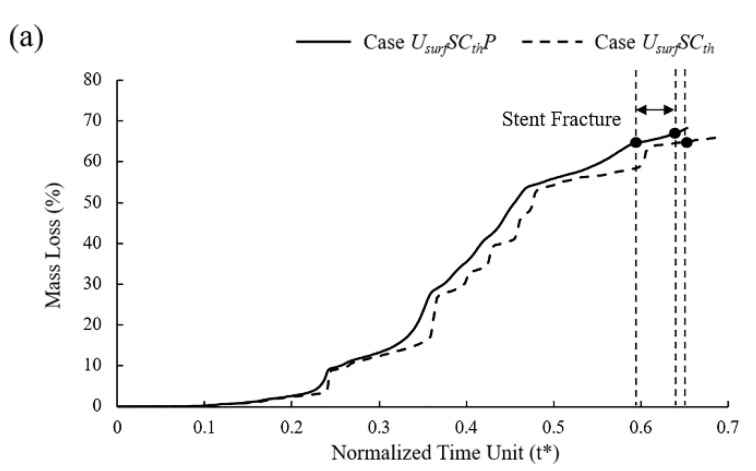

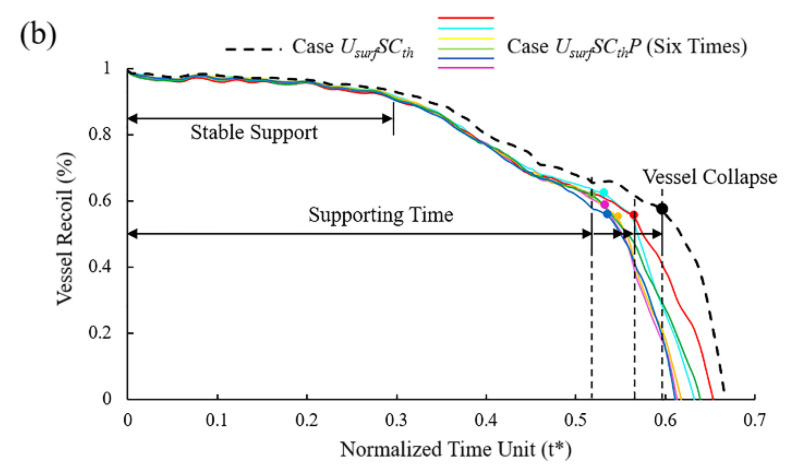
(**a**) Mass loss of Case *U_surf_SC_th_P* and Case *U_surf_SC_th_* during degradation; (**b**) vessel recoil due to loss of mechanical strength of stents during degradation.

In six different sets of simulations, the mass loss curves remained almost consistent despite some differences in the degradation due to the pitting corrosion parameters assigned to the surface elements being the same with different locations (Figure 10a). Compared to the model without pitting corrosion, the degradation was accelerated to a certain extent regardless of the locations of pitting corrosion, and the time of stent fracture was in t* = 0.58~0.64.

In the initial stage of stent degradation, there was a stable effective support time when the vessel diameter hardly changed (Figure 10b). After t* = 0.3, the diameter of the vessel began to change significantly from the increase in stent mass loss after the struts become thinner. It is still considered that the stent could provide effective support for the vessel in this stage. There would be a sudden loss of support at a certain moment around t* = 0.52~0.57, and the vessel would collapse before the stent fracture. No matter how the pitting corrosion is distributed, the supporting performance and support time were reduced compared to Case *U_surf_SC_th_*. The supporting time was reduced by up to 12% when pitting corrosion was located in the critical location of the stent. Before the vessel collapse, the stent could provide effective support for the vessel with the diameter difference reaching more than 60% before and after stent implantation.

## 4. Discussion

In this study, an FEA model based on the CDM principle was established to describe the degradation process and service performance of the BVSs by the phenomenological description of the damage. We made several improvements to the degradation model by considering the combined effect of multiple corrosion factors to explore a more realistic non-uniform degradation process.

The corrosion rate of the element was dynamically adjusted according to the number of surfaces of the element exposed to the corrosive environment during degradation. The results indicated that the elements with more exposed surfaces at the edges of the stent degraded first, and the cross-section of the stent gradually became round from square (Figure 4). It also smoothened the edges and corners that emerged during the degradation. This phenomenon is consistent with the work of Grogan et al. [43]. The proposed model can successfully capture the accelerated degradation of elements at the edges and corners of the stent under multi-dimensional attack [25]. The relationship between multi-dimension corrosions and mechanical property is reflected in this model. The multi-dimension corrosions increased the corrosion rates, and then reduced mechanical strength. During degradation, the number of exposed surfaces changed dynamically, especially the elements in corroded areas with poor mechanical integrity have more exposed surfaces and are more vulnerable to corrosion attack. Adjusting a higher corrosion rate for elements with more exposed surfaces, i.e., those at the edges and corners, helps to make the simulated degradation more realistic [44,45].

Stress corrosion plays a vital role in the degradation process [12]. The structure may fail under the safe load due to the mutual promotion of electrochemical corrosion in the corrosive environment and the mechanical damage under stress. Stress corrosion is the dominant factor affecting element removal at the beginning of the degradation. The elements subject to stress corrosion are mainly concentrated in the high tensile stress area at the corolla (Figure 7). These areas are critical in affecting the supporting performance, despite its small area on the stent. As mentioned in work by Li et al. [46], the stress corrosion threshold affected the corrosion rate, and thus stress threshold was an essential parameter for the degradation process. It is necessary to consider the stress threshold in more detail, such as the dynamic changes with the degradation progress. The dynamic adjustment of the stress threshold changes the degradation morphology and accelerates the degradation of the stent. This work might be helpful for studying the problem of non-uniform degradation and early failure after stent implantation [16].

In this research, the ratio of pitting corrosion to uniform corrosion was controlled by limiting the range of pitting corrosion parameters [13]. As a kind of local corrosion, pitting corrosion is mainly affected by the defect location during stent production and implantation. The above two factors can be controlled in the current production process; thus, pitting corrosion will not become the dominant factor for the degradation. This work confirmed that the pitting corrosion occurs independently during degradation and is unaffected by either the stress state or the material composition [43]. However, pitting corrosion plays a significant role in accelerating the local fracture of the stent compared with critical locations without pitting corrosion (Figure 9). Pitting corrosion in high-stress regions changes the shape of the stent, and the resulting stress concentration accelerates the development of stress corrosion. Therefore, it is necessary to consider the pitting corrosion effect in the degradation of the BVSs.

Stent degradation is a complex process in which many factors interact with each other. As consistent with previous work [44], the material properties of the stents are continuously weakened, and the geometry of the struts is gradually changed with the degradation. The mechanical strength of the stent decreases gradually with the decrease in the remaining volume (Figure 10b). Most of the mass loss of the stent comes from uniform corrosion, while the local stress corrosion and pitting corrosion plays a more critical role in the service life of the stent. As can be seen, the stent degrades rapidly after a stable support time [25]. The stents would suddenly lose support to the vessel and lead the vessel to collapse at one moment in the degradation process. However, we found that this moment precedes when the stent loses its mechanical integrity. Therefore, the effective support time of the BVSs should adapt to the vascular remodeling time to prevent adverse events such as the sudden collapse of the vessel.

Some limitations might exist in this study. Only blood was considered as the corrosive environment in this paper, while the vessel wall is also one. The corrosion rate of the elements only in contact with the vessel wall was temporarily set to be 0 due to the lack of the corrosion rate in the corrosive environment of the vessel wall, which is lower in the absence of blood flow [22]. Both corrosive environments will be considered in future work, and the corrosion rate needs to be differentiated. Especially for locations with stent malapposition, the stent is separated from the vessel wall and suspended in the blood. The outer surface of the struts is completely exposed to the blood, which might be more likely to fracture than the case where the stent is in contact with the vessel wall. This method might be helpful for exploring the degradation and fracture of the BVSs with malapposition. We will calibrate the model by experiments in future work to obtain the degradation morphology in more detail and predict the service time of the stent more accurately.

## 5. Conclusions

In this paper, a BVS non-uniform degradation model was established considering the combined action of multiple factors. The results demonstrated that both the increase in the number of exposed surfaces of the element and the change in the stress corrosion threshold expedited BVSs’ degradation process compared with the uniform degradation model. In particular, the addition of the pitting corrosion model significantly affected the service life of BVSs. The analysis proved that the stent could effectively support for the vessel by reaching more than 60% treatment effect during service. The phenomena of the numerical simulation results with the proposed model were more consistent with the real degradation scenario than the commonly used models. The corrosion rate was adjusted based on hemodynamic results that will be considered in future work to optimize the present model, especially in the case of stent malapposition. This work might provide a numerical simulation method and scientific basis for performance evaluation, the optimization of structural design, and the development of other alloys for BVSs.

## Figures and Tables

**Table 1:** Parameters for the stent degradation model [17,18,19].

Parameters	$\delta_{U}$	$k_{U}$	$\delta_{\mathrm{SC}}$	*S*	*R*	$\sigma_{\mathrm{th}}$	*β*
Value	0.1 mm	0.05/h	0.07 mm	$0.005\;{\mathrm{mm}}^{2}\sqrt{h\;}$/N	2	66 MPa	0.8

**Table 2:** Material properties [17].

Part	Density (g/cm^3^)	Young’s Modulus (MPa)	Poisson’s Ratio	Yield Stress (MPa)
Stent	8.5	74,300 (Max)~300 (Min)	0.3	220
Artery	1.12	-	-	-
Balloon	1.256	920	0.4	-

## Data Availability

Not applicable.
